# Nonlocal metasurfaces: universal modal maps governed by a nonlocal generalized Snell’s law

**DOI:** 10.1515/nanoph-2025-0051

**Published:** 2025-05-22

**Authors:** Adam Overvig, Francesco Monticone

**Affiliations:** Department of Physics, Stevens Institute of Technology, Hoboken, NJ, 07030, USA; School of Electrical and Computer Engineering, 5922 Cornell University, Ithaca, NY, 14853, USA

**Keywords:** metasurfaces, metamaterials, nonlocality, nanophotonics, nonlocal flat optics

## Abstract

In this opinion, we describe the potential of an emerging class of flat optics known as “nonlocal metasurfaces” to manipulate light in both real space and momentum space. While the ultimate form of a conventional “local” metasurface can be viewed as a universal generator of any desired waveform from a fixed input wavefront, the ultimate form of a nonlocal metasurface would instead act as a universal “map” from a given set of input waveforms to a set of orthogonal output waveforms. Here, we discuss how this implies four-dimensional information capacity, drastically enhancing information density compared to local metasurfaces. We discuss a framework using scattering matrices and a nonlocal generalized Snell’s law to describe nonlocal metasurfaces. We comment on the potential, progress, and practicality of this ambitious vision, suggesting limitations and next steps.

## 1 Introduction

Metasurfaces (MS) and photonic crystal slabs (PC) are classes of flat optical components with comparable form yet complementary function. MS are most closely associated with subwavelength arrays of spatially varying fill factor (e.g., shapes and sizes of constituent elements) patterned in high-index thin films or plasmonic features, with the goal of spatially varying the properties of scattered light [1], [2]. PC are principally engineered by their array factor (that is, how a few identical elements are placed in relation to one another) in order to control the momentum properties of light [3], [4]. They are known for broadband features such as photonic bandgaps [5] or reflection bands [6], as well as sharp spectral features called Fano resonances with long optical lifetimes (Q-factors) [7]. Therefore, MS are most closely associated with spatial properties of the wavefront, while PC are most closely associated with momentum properties of the wavefront. Both enable flat, lightweight, and compact systems compatible with mass-manufacturing.

Nonlocal metasurfaces (NMS) are an emerging class seeking to combine both fill and array factor engineering [8], [9]. “Nonlocality”, here, refers to the scattering of light due to many adjacent elements. “Locality”, on the other hand, is the conventional approximation employed in MS and effective media optics ignoring such effects (where they are seen as a nuisance). NMS aim to leverage interelement coupling in order to surpass limitations of LMS, and come in two broad categories [8], which we label as “momentum” NMS (M-NMS) and “spatial” NMS (S-NMS). M-NMS customize plane wave responses as a function of incident angle; they commonly take the form of customized PC, gratings, or thin film stacks, and are typically subwavelength in period. Attractive applications include optical computing [10], edge detection [11], and compression of space in so-called “spaceplates” [12], [13], [14]. S-NMS, on the other hand, are spatially varying, often aperiodically. They typically take the form of perturbed photonic crystal slabs or gratings, and are designed to shape light both spatially and spectrally [15], [16], [17], [18]. The physics and symmetry-based design principles of bound and quasi-bound states in the continuum, abbreviated as BICs [19], [20] and q-BICs [21], [22], respectively, feature heavily in both M-NMS and S-NMS.

Here, we compare related classes of local and nonlocal flat optics and provide our opinion on how to frame the full potential of NMS: while the ultimate LMS can be viewed as a “universal wavefront generator”, the ultimate S-NMS can be viewed as a “universal modal map”. We argue that S-NMS further generalize the “Generalized Snell’s law”, a foundational concept of LMS [1]. This “Nonlocal generalized Snell’s law” clarifies how S-NMS offer higher dimensionality in information encoding compared to LMS. We briefly review progress towards this vision, and point out opportunities for the future, with the hope to guide future efforts towards S-NMS with greatly increased information capacity. We emphasize that there exist many open questions on both the practicality and physicality of this vision of the ultimate nonlocal metasurface – realistic implementations of S-NMS systems may fall short of the version described here. We are agnostic regarding what will be realized in practice – rather, our intention is to provide a point of view within which highly multi-functional meta-topics may be developed and bounds and constraints involving reciprocity, bandwidth, thickness, and refractive index, may be studied. Advancements in this area promise a new frontier for manipulating light with next-generation optics.

## 2 The potential of nonlocal metasurfaces

**Figure 1:**
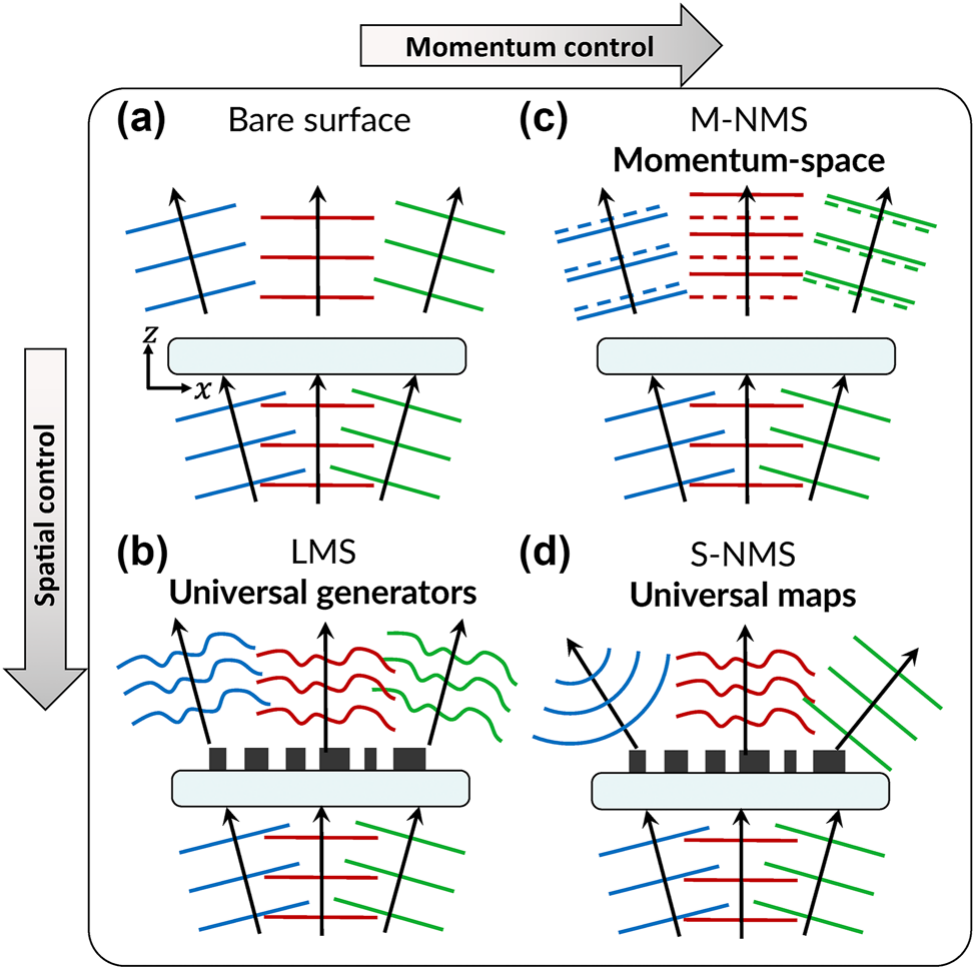
Control of light in bare, local, and nonlocal metasurfaces. (a) A bare surface scatters three plane waves without independent control thereof. (b) The “ultimate form” of a local metasurface can be viewed as a universal generator for a given incident plane wave – nearby momenta produce shifted copies of the generated waveform. (c) An M-NMS can be viewed as the analogue of a local metasurface but in momentum-space, customizing each plane wave but leaving them spatially unpatterned. (d) In its “ultimate form”, an S-NMS acts as a universal modal map.

To clarify the potential of nonlocal metasurfaces as generalized flat optical media, we contrast bare surfaces [Figure 1a], LMS [Figure 1b], M-NMS [Figure 1c] and S-NMS [Figure 1d]. First, a bare interface serves as a simple reference case: planewaves respond according to Snell’s law. That is, they pass without altering their lateral momentum (conservation of momentum) and with minimal distinction according to frequency (except for dispersion in the refractive indices $n\left(\lambda \right)$ , which we ignore here for simplicity). A typical response is represented by the red, green, and blue waves in Figure 1a, which should be understood as portraying variance in incident angle and/or frequency.

Second, an LMS may be considered idealized complex transparencies, i.e., thin film holograms with subwavelength pixels. Their design procedure typically comprises computational search and optimization of a “library” of local geometries (called “meta-units”) with widely varying scattering properties. The library elements serve as building blocks for a rational design scheme of assigning geometries across MS’s aperture according to the required scattering. For a transmissive device, the required scattering typically comprises four degrees of freedom (DoF): the amplitude *A*, phase Φ, polarization angle *ψ*, and polarization ellipticity *χ*. A metasurface library with such control is a “universal wavefront generator” [Figure 1b]: from an unpatterned beam, any physical complex vectorial wavefront may be produced: $\left(A\left(r\right),\Phi \left(r\right),\psi \left(r\right),\chi \left(r\right)\right)$ . However, due to the near-locality of their operation, their response is necessarily broad in momentum, conferring relatively large bandwidth and angular tolerance: nearby momenta will approximately map to the same generated wavefront.

To reach beyond the viewpoint of LMS, we incorporate nonlocality. However, we note that, in principle, a transmissive LMS may have eight DoF: there are eight free parameters in a two-by-two complex Jones matrix, amounting to simultaneous control of the output waves for two orthogonal input polarizations. This suggests a view of flat optics as “maps” of multiple input states to any desired output states. For LMS, this usually amounts to distinct polarizations mapping to distinct output states [23], but careful design can yield multiple independent phase-only holograms at distinct incident angles [24], presaging NMS.

Third, M-NMS represent the generalization of a bare surface response in momentum space rather than real space [Figure 1c]: the amplitude, phase, and polarization state can be shaped as a function of incident angle and/or frequency. For instance, Figure 1c depicts the customization of three incoming plane waves, which emerge as plane waves but with three distinct custom phase delays. The ultimate M-NMS therefore, would have “pointwise” control over planewaves in such a fashion, $\left(A\left(k\right),\Phi \left(k\right),\psi \left(k\right),\chi \left(k\right)\right)$ . However, many applications of interest need only smoothly varying changes as a function of angle [8].

Finally, S-NMS [Figure 1d] complete the generalization by imparting distinct spatial profiles for each incident mode: the amplitude, phase, and polarization may be shaped spatially for multiple incoming plane waves, amounting to the control $\left(A\left(r,k\right),\Phi \left(r,k\right),\psi \left(r,k\right),\chi \left(r,k\right)\right)$ . This control is comparable to the concept from ray optics called a “Lightfield” $L\left(x,y,k_{x},k_{y}\right)$ [25]: i.e., while LMS and M-NMS both encode two-dimensional information (in real space $r=\left(x,y\right)$ and momentum space $k=\left(k_{x},k_{y}\right)$ , respectively), the ultimate S-NMS is four-dimensional. Naturally, such an operation is limited by reciprocity: the outgoing waveforms for each incident plane wave must be mutually orthogonal. Therefore, not all choices of $\left(A\left(r,k\right),\Phi \left(r,k\right),\psi \left(r,k\right),\chi \left(r,k\right)\right)$ are physically possible in a reciprocal system. As a simple example: if two, different incident plane waves could create the *same* outgoing wave with unity efficiency, its reversal would create an unphysical ambiguity regarding which plane wave is recovered.

To properly frame the capabilities of S-NMS, we may decompose the incoming and outgoing wavefronts into two orthogonal, complete basis sets, consistent with the “modal view” of optics [26]. Then, the ultimate nonlocal metasurface takes the form of a “Universal Modal Map” *S*_*map*_, scattering each waveform in a chosen set of incoming basis waveforms *ψ*_*in*_, to a second set of outgoing basis waveforms *ψ*_*out*_: *ψ*_*out*_ = *S*_*map*_ *ψ*_*in*_. In the example in Figure 1d, the chosen inputs *ψ*_*in*_ are plane waves, while the outputs *ψ*_*out*_ are customized wavefronts. This represents one example choice: That is, while a given S-NMS only maps one basis to another basis (i.e., implements a single scattering matrix), the complete set of all S-NMS comprises the mapping of any bases: i.e., the rational specification of any *S*_*map*_. Similarly, while a given LMS only produces one output wavefront, the complete set of all LMS comprises the generation of any desired output wavefront. In brief, the difference between LMS and S-NMS can be summarized as follows: while “universal generators” (LMS) can take one chosen input to a desired output (one to one mapping), “universal maps” (S-NMS) can take *many* chosen inputs to *many* desired outputs (many to many mapping).

## 3 Physics beyond the generalized Snell’s law

While LMS functionalities have been studied for decades [27], they gained significant attention within the framework of the “Generalized Snell’s law” [1]. This viewpoint applies to only a subset of functionalities, namely, to smoothly varying phase profiles and to scalar diffraction (i.e., ignoring polarization). Despite this limitation, such a framework is highly clarifying. That is, Snell’s law:

(1) $$n_{2}\sin\left(\theta_{2}\right)=n_{1}\sin\left(\theta_{1}\right)$$

for incident and outgoing angles *θ*_1_ and *θ*_2_ involving refractive indices *n*_1_ and *n*_2_, is altered by the local scattering phase $\Phi \left(x\right)$ to become the generalized Snell’s law [1]:

(2) $$n_{2}\sin\left(\theta_{2}\right)=n_{1}\sin\left(\theta_{1}\right)+\frac{1}{k_{0}}\nabla \Phi ,$$

where $k_{0}=\frac{2\pi }{\lambda }$ is the wavevector. Here, we consider one-dimensional devices varying along a spatial dimension *x* for simplicity; we also focus our discussion on monochromatic waves, ignoring material dispersion in the refractive indices *n*_1_ and *n*_2_ and dispersion of the efficiency of the phase gradient in a metasurface. Moreover, for a smoothly varying phase function $\Phi \left(x\right)$ , it is typical to consider each local region of an MS to act as a beam deflector characterized by ∇Φ and consider the beam deflection angle to vary across the aperture. A metasurface lens is a prototypical example: the locus of the beam deflection angles is the focal spot. Hence, even in aperiodic systems, an LMS can often be understood in terms of periodic scattering. In this section, we leverage this fact to compare LMS to NMS.

### 3.1 Scattering matrices and the nonlocal generalized Snell’s law

To clarify the distinction between metasurface categories, we construct a scattering matrix $S_{ij}\left(k_{i},k_{j}^{\prime }\right)$ for a periodic device with period *P* having diffraction orders indexed by the integer *m*. The entries are complex numbers quantifying the amplitude and phase of scattering from side *j* to side *i* as a function of incident momentum $k_{j}^{\prime }$ and outgoing momentum *k*_*i*_. Throughout, primed coordinates refer to incident conditions, while unprimed refer to outgoing conditions. By conservation of quasi-momentum (i.e., the grating equation), only certain combination of $k_{j}^{\prime }$ and *k*_*i*_ yield nonzero scattering. We may enforce this with a delta function $\delta \left[f_{m}\left(k_{i},k_{j}^{\prime }\right)\right]$ , where the argument $f_{m}\left(k_{i},k_{j}^{\prime }\right)$ enforces conservation of momentum for the *m*th diffraction order:

(3) $$f_{m}\left(k_{i},k_{j}^{\prime }\right)=k_{i}-k_{j}^{\prime }-m\frac{2\pi }{P}.$$

Then, we allow the scattering matrix to have any complex amplitude coefficient $a_{m}\left(k_{i},k_{j}^{\prime }\right)$ for a given incoming momentum $k_{j}^{\prime }$ connected to an outgoing momentum *k*_*i*_ by a diffraction order *m*. Together, we arrive at the following form for a thin film periodic scatterer:

(4) $$S_{ij}\left(k_{i},k_{j}^{\prime }\right)=\underset{m}{\sum }a_{m}\left(k_{i},k_{j}^{\prime }\right)\delta \left[f_{m}\left(k_{i},k_{j}^{\prime }\right)\right].$$

When discretized into *N*_*k*_ momenta, $S_{ij}\left(k_{i},k_{j}^{\prime }\right)$ is a 2*N*_*k*_ × 2*N*_*k*_ matrix for a for a two-sided metasurface, and the delta function is the Kronecker delta, yielding an entry of 1 when its argument is 0, and an entry of 0 otherwise. The incoming and outgoing fields are organized as

(5) $$E_{in}=\left[\begin{array}{c} E_{in,1}\left(k_{1}^{\prime }\right)\\ E_{in,2}\left(k_{2}^{\prime }\right) \end{array}\right],\;E_{\mathrm{out}}=\left[\begin{array}{c} E_{\mathrm{out,1}}\left(k_{1}\right)\\ E_{\mathrm{out,2}}\left(k_{2}\right) \end{array}\right]$$

for sides 1 and 2, respectively. For instance, $E_{in,1}\left(k^{\prime }\right)$ is a column vector with *N*_*k*_ elements, where each entry is the complex amplitude of the plane wave of the corresponding incoming wavevector $k_{1}^{\prime }$ . The wavenumbers are lateral momenta, e.g., $k_{1}^{\prime }=n_{1}k_{0}\sin\left(\theta_{in}\right)$ . Then, the scattering matrix is constructed as

(6) $$S=\left[\begin{array}{cc} S_{11}\left(k_{1},k_{1}^{\prime }\right) & S_{12}\left(k_{1},k_{2}^{\prime }\right)\\ S_{21}\left(k_{2},k_{1}^{\prime }\right) & S_{22}\left(k_{2},k_{2}^{\prime }\right) \end{array}\right].$$

Where each submatrix is *N*_*k*_ × *N*_*k*_. Finally, the outgoing field is then given by

(7) $$E_{\mathrm{out}}=SE_{in}.$$

The nature of each of the four device categories in Figure 1 can be clarified by how they manipulate the elements of this scattering matrix. First, for a bare interface, *a*_*m*_ are the Fresnel coefficients, which vary slowly as a function of $k_{j}^{\prime }$ (we ignore this variance here for simplicity). Yet only the *m* = 0 diffraction order is possible, leaving only *a*_0_ nonzero; the grating equation becomes Snell’s law. This is enforced by the argument of the delta function, which yields scattering only when outgoing momentum is equal to incoming momentum. Namely,

(8) $$S_{ij}\left(k_{i},k_{j}^{\prime }\right)=a_{0}\delta \left[k_{i}-k_{j}^{\prime }\right].$$

Second, LMS can be considered diffractive elements that select *m* = ±1 by implementing a linear phase gradient $\nabla \Phi =\pm \frac{2\pi }{P}$ . The coefficients *a*_±1_ are the complex amplitude of the diffracted wave. The result is scattering governed by the generalized Snell’s law:

(9) $$S_{ij}\left(k_{i},k_{j}^{\prime }\right)=a_{\pm 1}\delta \left[k_{i}-k_{j}^{\prime }\mp \nabla \Phi \right].$$

Note: beyond smoothly varying phase profiles, more than just a single diffraction order can be picked out, in which case *a*_*m*_ encode the amplitude and phase into each order.

Third, typically M-NMS are implemented as 0*th* order diffraction gratings that satisfy Snell’s law (i.e., $k_{i}=k_{j}^{\prime }$ ) but with customized, sharply varying coefficients beyond Fresnel coefficients, i.e.,

(10) $$a_{0}\left(k_{j}^{\prime },k_{j}^{\prime }\right)=h\left(k_{j}^{\prime }\right)$$

for some chosen function $h\left(k_{j}^{\prime }\right)$ . Namely,

(11) $$S_{ij}\left(k_{i},k_{j}^{\prime }\right)=h\left(k_{j}^{\prime }\right)\delta \left[k_{i}-k_{j}^{\prime }\right].$$

Finally, S-NMS provide generalized control over each aspect of the scattering equation. That is, similar to M-NMS, S-NMS may control the momentum dependence of the response, but now for each coefficient *a*_*m*_. Meanwhile, like LMS they are not limited to Snell’s law. As a consequence, the effective phase gradient can depend on which side of the interface the incident beam arrives and may vary as a function of incident angle [28]. We have the general form

(12) $$S_{ij}\left(k_{i},k_{j}^{\prime }\right)=\underset{m}{\sum }a_{m}\left(k_{i},k_{j}^{\prime }\right)\delta \left[k_{i}-k_{j}^{\prime }-\nabla \Phi_{ij}\left(k_{j}^{\prime }\right)\right].$$

Here, the argument is the nonlocal generalized Snell’s law, allowing scattering only when

(13) $$k_{i}=k_{j}^{\prime }+\nabla \Phi_{ij}\left(k_{j}^{\prime }\right).$$

Or, writing $k_{i}=n_{\mathrm{out}}k_{0}\sin\left(\theta_{\mathrm{out}}\right)$ and $k_{j}^{\prime }=n_{in}k_{0}\sin\left(\theta_{in}\right)$ , and considering only transmission from one side,

(14) $$n_{\mathrm{out}}\sin\left(\theta_{\mathrm{out}}\right)=n_{in}\sin\left(\theta_{in}\right)+\frac{1}{k_{0}}\nabla \Phi \left(\theta_{in}\right).$$

Notably, not any nonlocal phase gradient $\nabla \Phi_{ij}\left(k_{j}^{\prime }\right)$ is physical in a reciprocal device. By reciprocity, the scattering matrix must be symmetric. For a pair of momenta *k*_*a*_ and *k*_*b*_:

(15) $$S_{ij}\left(k_{a},k_{b}\right)=S_{ji}\left(-k_{b},-k_{a}\right).$$

This enforces the condition:

(16) $$\nabla \Phi_{ij}\left(k_{b}\right)=\nabla \Phi_{ji}\left(-k_{a}\right)$$

which, together with the delta function, guarantees that light incident angle momentum *k*_*b*_ from side *j* and diffracted to outgoing momentum *k*_*a*_ on side *i*, is equivalently diffracted when reversed: diffracting from momentum −*k*_*a*_ from side *i* to −*k*_*b*_ and side *j*. Given the dependence of incident side and outgoing side, the nonlocal generalized Snell’s law is best considered in the context of the scattering matrix, rather than as a standalone condition.

Notably, S-NMS may be implemented with several individually customized q-BICs, which we index by *q*. When mediated by a single q-BIC the scattering typically picks out *m* = ±2 [15] due to coupling and in and out via a geometric phase, ∇Φ_*ij*,*q*_. Hence the scattering due to such a q-BIC has the form

(17) $$S_{ij}\left(k_{i},k_{j}^{\prime }\right)=a_{\pm 2}\delta \left[k_{i}-k_{j}^{\prime }\mp \nabla \Phi_{ij,q}\right].$$

For *M* q-BICs, we have

(18) $$S_{ij}\left(k_{i},k_{j}^{\prime }\right)=\overset{M}{\underset{q=1}{\sum }}a_{q}\delta \left[k_{i}-k_{j}^{\prime }-\nabla \Phi_{ij,q}\right].$$

Since each q-BIC may have a custom dispersion relation, *a*_*q*_ is associated with distinct incident momentum, and we may use this scheme to build a general S-NMS by use of multi-perturbation [15] and/or cascaded sets of many multi-functional S-NMS [17].

### 3.2 Examples in reflective phase gradient devices

**Figure 2:**
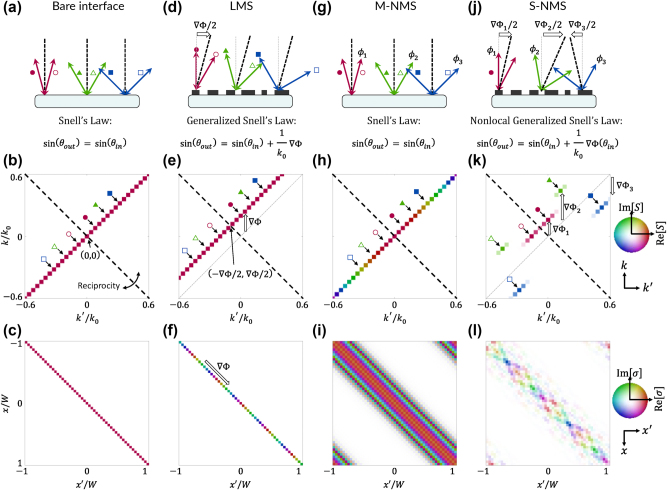
Comparison of reflective scattering in four periodic thin film systems. (a) Specular reflection at a bare interface, shown via three example reciprocal pairs of rays (tracked with open and closed colored markers). Corresponding k-space scattering matrix (b) and real-space scattering matrix (c). (d) Anomalous reflection due to an LMS, shown via three example reciprocal pairs of rays. (e) Corresponding k-space scattering matrix $S\left(k,k\prime \right)$ and (f) real-space scattering matrix $\sigma \left(x,x\prime \right)$ (f). (g) Plane wave customization in an M-NMS, shown via three example reciprocal pairs of rays scattering with three phase values *ϕ*_1_, *ϕ*_2_, *ϕ*_3_. (h) Corresponding k-space scattering matrix $S\left(k,k\prime \right)$ and (i) real-space scattering matrix $\sigma \left(x,x\prime \right)$ . (j) Nonlocal anomalous reflection in an S-NMS, shown via three example reciprocal pairs of rays scattering with three deflection angles and three phase values *ϕ*_1_, *ϕ*_2_, *ϕ*_3_. Corresponding k-space scattering matrix $S\left(k,k\prime \right)$ (k) and real-space scattering matrix $\sigma \left(x,x\prime \right)$ (l). Note that in all scattering matrices, saturation tracks magnitude and hue tracks phase of each element. Primed coordinates *x*′ and *k*′ on the horizontal axes refer to inputs while unprimed coordinates *x* and *k* on the vertical axes refer to outputs.

To gain further insight into these scattering equations and the role of reciprocity, we consider the simple case of scalar diffraction in reflection: i.e., we study $S_{11}\left(k,k^{\prime }\right)$ . Here, the refractive index of the incident medium (which is also the outgoing medium) can be considered unity without loss of generality. First, we again consider the bare interface as a reference case. Snell’s law is simply the law of reflection; reciprocity may be considered as the requirement of a symmetric response for pairs of incident momenta mirrored across the device normal [Figure 2a]. Visualizing the scattering matrix for this case [Figure 2b], we have a sparse matrix composed of all zeros except the diagonal where *k* = *k*′. The three example pairs of rays in Figure 2a are marked, showing that they are mirror copies of each other when reflected over the 45° line. While seemingly trivial in this case, we note that the coordinate $\left(0,0\right)$ is the only momentum pair on the allowed diffraction order that also intersects the reciprocity line. That is, normal incidence naturally bifurcates the scattering matrix; it is the “fulcrum” about which the scattering is balanced by reciprocity [marked by the black dashed lines in Figure 2a]. Meanwhile, the hue represents the variance of the coefficients *a*_*m*_. Here, each entry of the diagonal is the same color, signifying no customization of each planewave. We are also interested in the scattering matrix in real space, obtained by a mixed Fourier transform as a function of input position *x*′ and output position *x* [28]:

(19) $$\sigma \left(x,x^{\prime }\right)=F_{k}\left\{F_{k^{\prime }}^{-1}\left\{S\left(k,k^{\prime }\right)\right\}\right\},$$

where $F_{k}$ is a Fourier transform mapping *k* to *x* and $F_{k^{\prime }}^{-1}$ is an inverse Fourier transform mapping *k*′ to *x*′. For the bare interface, we see in Figure 2c that only entries such that *x* = *x*′ are nonzero, consistent with locality, while as expected they each have the same phase.

Second, we consider LMS with a phase gradient in the positive *x* direction [Figure 2d]. Light incident at *k*′ = 0 is diffracted to *k* = ∇Φ. At the precise incident condition *k*′ = −∇Φ/2, the outgoing wave is retroreflected: *k* = *k*′ + ∇Φ = −*k*′, marked by the black dashed lines in Figure 2d. Every other ray comes as a pair that is symmetric about the retroreflection condition [shown as red, green, and blue in Figure 2d]. We see in Figure 2e the scattering matrix is simply a shifted diagonal corresponding to the *m* = 1 diffraction order. Only along this line is a nonzero scattering event (satisfying conservation of momentum as enforced by the generalized Snell’s law). The intersection of this line and the reciprocity line is precisely the retroreflected pair $\left(-\nabla \Phi /2,\nabla \Phi /2\right)$ , the fulcrum for reciprocity in this more general case. Note that without a phase gradient, specular reflection and retroreflection are both satisfied at *k*′ = 0. Hence, the “generalization” of the generalized Snell’s law can be considered the introduction of a “tilt” to the retroflected rays. Finally, the scattering matrix in real space, $\sigma \left(x,x^{\prime }\right)$ , is shown in Figure 2f, where again we see a purely local response (nonzero entries only when *x* = *x*′) but, this time, with a phase that varies along the main diagonal (the phase gradient). Notably, due to the diagonal form of this 2D matrix, the scattering problem can – and conventionally is – treated as 1D (or in the case of an *xy* metasurface, 4D reduces to 2D).

Third, we consider M-NMS. As discussed above, the conventional Snell’s law is satisfied: momenta are paired by reciprocity such that they are symmetric about the device normal. However, here they scatter with some custom phase $\phi \left(k^{\prime }\right)=\phi \left(-k^{\prime }\right)$ [Figure 2g]. Figure 2h shows an example scattering matrix with the smoothly varying phase of a spaceplate $\Phi \left(k^{\prime }\right)=5.5{\left(k^{\prime }/k_{0}\right)}^{2}$ . The corresponding real space scattering matrix is shown in Figure 2i, where we see a symmetric, but *not* diagonal matrix. The off-diagonal matrix elements confer nonlocality – nonzero correlation between positions *x*′ and distant positions *x* ≠ *x*′. Hence, in contrast to local responses, each spatial coordinate must be considered twice, doubling the dimensionality of the scattering behavior compared to the real-space dimensionality of the device. However, while the scattering matrix varies in the off-diagonal direction, it is spatially invariant along the main diagonal – there is no spatial control. As a result, we have a shift-invariant kernel dependent on only the difference *x* − *x*′ rather than *x* and *x*′ independently. The convolution theorem therefore allows study of these devices purely in *k* space, reducing the dimensionality of the problem back down to one (or in the case of an *xy* metasurface, to two).

Finally, we consider S-NMS, amounting to the full generalization of reciprocal periodic thin film scattering. Figure 2j depicts the freedom of the nonlocal generalized Snell’s law subject to reciprocity: the three momentum pairs scatter to and from each other as reciprocal copies about three distinct fulcra associated with phase gradients ∇Φ_1_, ∇Φ_2_, and ∇Φ_3_. Moreover, as in M-NMS, a distinct set of phases may be implemented to each pair: *ϕ*_1_, *ϕ*_2_, and *ϕ*_3_. The resulting scattering matrix is shown in Figure 2k, where we see the three scattering events forming symmetric pairs about the reciprocal line, but associated with distinct diffraction orders according to the phase gradients. Hence, the nonlocal generalized Snell’s law further generalizes Snell’s law by allowing a plurality of retroreflection angles about which responses are symmetric by reciprocity. The corresponding real space scattering matrix in Figure 2l can be seen to be approaching a general full matrix (albeit symmetric, due to reciprocity). Like LMS, the properties vary along in the direction parallel to the main diagonal; yet, like M-NMS, they *also* vary in the direction perpendicular to the main diagonal. For this reason, neither the reduction of dimensionality of the scattering problem for LMS (due to locality) nor M-NMS (shift invariance) apply; this general scattering behavior cannot be fully treated in solely real space or solely in momentum space – it is four dimensional for a two-dimensional surface.

## 4 Progress and barriers toward this vision

The ultimate form of S-NMS as universal modal maps amounts to an incredible increase in information density encoded into a metasurface, which has not been achieved to date. Still, recent progress towards these goals has been notable, suggesting future pathways. Notably, diffractive NMS [9] offer a rational design framework for implementing an individual mapping of the sort seen in Figure 2j. By spatially customizing the selection rules of a q-BIC, anomalous reflection or transmission is achievable with a high degree of selectivity to incident angle and frequency [22]. In Ref. [15], it was argued that up to four frequencies per metasurface can be encoded simultaneously by using multiple orthogonal perturbations; up to three were demonstrated numerically. In Ref. [17], such a scheme was experimentally demonstrated with up to two functionalities per metasurface. Moreover, it was shown that the mutual transparency of multiple metasurfaces may be leveraged to cascade functionalities, achieving up to four frequencies simultaneously. Given the dispersion of these modes, the same approaches may be used to implement multi-angle operation instead of multi-frequency operation. Meanwhile, spatio-temporal coupled mode theory [28] has been developed as a simple mathematical framework with remarkably successful capture of both local and nonlocal properties of these devices. Currently, the theory is limited to single q-BICs and to parabolic bands, but coupled mode theories can be generalized to multiple modes [29] and linear dispersion [30] as well. Using such a tool, scattering matrices in k-space and real space, which have proven highly useful in describing the required nonlocality for a desired functionality [31], [32], may be readily computed based on a discrete set of guided modes. Therefore, the symmetry-controlled q-BIC platform appears ideally poised to make rapid progress in the direction of universal modal mapping.

Both computational and rational efforts can be fruitful for these endeavors. Notably, early efforts in Ref. [33] closely resemble the example chosen in Figure 2j, and were achieved via inverse design optimization. Inverse design approaches appear to generically achieve resonant, nonlocal responses if the response at other incident angles and/or frequencies is not constrained [34]. This suggests that computational optimization could be highly useful for specific functionalities, and advanced techniques in machine learning could effectively reduce the dimensionality of the relevant design space to reduce the complexity of the problem [35]. However, we emphasize that the “universality” in “universal modal maps” idealizes a rational design scheme akin to that in LMS; we desire a “library” of responses for rational configuration of any desired modal map, just as LMS are conventionally implemented using a “library” of structures for rational configuration of any desired generated wavefront. The selection rules governing q-BICs offer an “alphabet” of structures [22] to begin to construct such a library, especially when extended to multi-level structures [35], [36], [37]. But so far, compared to what is possible, only a small subset of the degrees of freedom possible have been accessed, with most efforts extending a dimer approach introduced in Ref. [15].

Several challenges and obstacles, some known but many still unknown, may cap the potential realization of the full vision of S-NMS as universal modal maps. We briefly comment on these issues. For example, it is increasingly clear that nonlocality is limited by thickness, bandwidth, and causality constraints [31], [32], [46], [47], [48]. For instance, a resonant response implies narrow bandwidth operation. Consider, for example, a mode with group velocity *v*_*g*_ and lifetime *τ*: the characteristic distance it travels laterally will be *ξ*_0_ ≈ *v*_*g*_ *τ*. Such a mode can correlate the responses at positions *x* and *x*′, but this effect becomes negligible when *x* − *x*′ ≫ *ξ*_0_. Hence, as the nonlocality grows, the required lifetime grows, and bandwidth narrows.

Most notably, a given universal modal map may require thick optical devices, amounting to a nonlocal “metamaterial” rather than a nonlocal “metasurface”. Intuitively, if we consider decomposing a map into individual pairs of planewaves as in Figure 2j, every customized pair that operates independently from the local response requires its own guided mode: to customize *M* planewaves, we require roughly *M* modes. In turn, we expect the required thickness to grow proportionally to the number of orthogonal guided modes *M*. These considerations have been formalized generically in terms of “overlapping nonlocality” in Ref. [32], suggesting the same conclusion: as the number of independent channels required for a certain optical function grows, so too does the required optical thickness. Put into terms of information density, the ultimate S-NMS encodes arbitrary correlations between each input position $\left(x^{\prime },y^{\prime }\right)$ and output position $\left(x,y\right)$ , and hence it scales as *N* ^2^ for a total number of meta-units *N*. In contrast, the information density for LMS or an M-NMS scales as *N*. However, for an S-NMS composed of many q-BIC metasurfaces, each component metasurface encodes *N* DoF per mode. Hence, the information density of the full S-NMS with *M* q-BICs scales as *MN*, requiring a number *M* = *N* of modes to truly realize an arbitrary functionality. Since *N* grows with lateral aperture size, and *M* grows with thickness, we intuitively expect a true universal meta-optic to be volumetric.

While the q-BIC implementation is particularly promising for implementing S-NMS, a few capabilities necessary for such a vision remain lacking. For a truly universal map, the ability to customize the outgoing wavefront must be achieved simultaneously with the ability to customize the incoming wavefront. However, so far, q-BIC metasurfaces have been limited by a limited form of wavefront selectivity: a specific wavefront will resonate, while other waveforms will not engage [37]. When losses are present, this selectivity has enabled advanced customization of thermal emission [49], [50]. Without losses, the outgoing wave is not independent of the selected incoming wave: it reflects as its *conjugate* [28], [37]. A fully universal modal map requires a q-BIC to selectively “catch” a desired wave, and then “release” a second desired wave. Progress towards this has been achieved varying the resonant frequency simultaneously with the phase profile [51], or moving the band-edge mode in momentum space [52] simultaneously with phase profile, suggesting this is a solvable problem in the near future. Along these same lines, it is highly desirable to be able to rationally design the degree of nonlocality (e.g., controlling dispersion between flat and linear bands [53]) while retaining local control over the scattering. Such a feat has recently been achieved at radiofrequencies by using metal vias [51], but has not been shown at optical frequencies and with dielectric materials. Generically, such advanced functionalities imply the requirement to break out-of-plane symmetries and reliably stack multiple layers. Recent progress on free-standing visible high-aspect ratio metasurfaces [54] suggest that with dedicated effort, such fabrication is achievable in the near future.

## 5 Summary and Outlook

As universal modal maps, S-NMS promise to vastly increase our command of wave-matter interactions. Beyond merely local devices, S-NMS *selectively* interact with light via resonances. Beyond being merely resonant devices, S-NMS *functionalize* resonances to impart transformations. They represent a general class of devices, with LMS and M-NMS representing subsets [9]. LMS have been enabling exciting applications in a compact form, such as imaging, holography, and spectropolarimetry [55]. Likewise, M-NMS have been enabling the compactification of functionalities previously considered the domain of 4f systems, such as edge detection and momentum-dependent wave manipulation [8], and exotic scattering effects such as unidirectional guided resonances [56]. As the generalization of both classes, S-NMS promise novel functionalities not possible in either class. New and emerging architectures may prove fruitful, such as vertically customized metasurfaces fabricated using many thin layers to comprise a single metasurface [57] or by combining the vertically stacked thin films associated with certain M-NMS [13] with in-plane patterned geometries associated with LMS and S-NMS. With advanced design, combined functionalities may be readily available, such as metalenses that simultaneously act as spaceplates, or metalenses that focus while also offering edge-detection capabilities. The momentum- and/or frequency-selectivity may uniquely enable certain forms of augmented reality combiners in thin films, a function conventionally requiring volume holograms (thick gratings) [16], [17]. And the inherently strong yet flexible wave-matter interactions in nonlocal and resonant optics suggest S-NMS as a powerful tool for next-generational reconfigurable, nonlinear, and quantum optics. Notably, we have constrained our discussion of S-NMS to what is possible with linear, passive, reciprocal media; going forward, active, time-varying, nonlinear, and/or nonreciprocal media may be incorporated into the design. A universal modal map represents the manipulation of waves to the fullest extent possible before the addition of exotic responses; the combination thereof promises unprecedented and exotic phenomena in compact, custom optics controlling the scattering, modulation, and absorption/emission of light.
